# Innate immunity and adjuvants

**DOI:** 10.1098/rstb.2011.0106

**Published:** 2011-10-12

**Authors:** Shizuo Akira

**Affiliations:** WPI Immunology Frontier Research Center, Osaka University, 3-1 Yamadaoka, Suita, Osaka 565-0871, Japan

**Keywords:** Toll-like receptor, signalling pathway, dendritic cell, influenza virus, interferon

## Abstract

Innate immunity was for a long time considered to be non-specific because the major function of this system is to digest pathogens and present antigens to the cells involved in acquired immunity. However, recent studies have shown that innate immunity is not non-specific, but is instead sufficiently specific to discriminate self from pathogens through evolutionarily conserved receptors, designated Toll-like receptors (TLRs). Indeed, innate immunity has a crucial role in early host defence against invading pathogens. Furthermore, TLRs were found to act as adjuvant receptors that create a bridge between innate and adaptive immunity, and to have important roles in the induction of adaptive immunity. This paradigm shift is now changing our thinking on the pathogenesis and treatment of infectious, immune and allergic diseases, as well as cancers. Besides TLRs, recent findings have revealed the presence of a cytosolic detector system for invading pathogens. I will review the mechanisms of pathogen recognition by TLRs and cytoplasmic receptors, and then discuss the roles of these receptors in the development of adaptive immunity in response to viral infection.

## 1. History of immunology

Vaccination was started by Edward Jenner based on the common observation that milkmaids who had suffered from cowpox were protected against smallpox: he used a cowpox vaccine to immunize humans against smallpox. In 1796, Jenner inoculated cowpox pus into the arm of a young boy. The boy became mildly ill with cowpox but soon recovered. Later, the boy was challenged with smallpox but showed no signs of infection. These findings showed that infection with cowpox could provide immunity against smallpox. The word ‘vaccination’, which was coined by Jenner for his treatment (from the Latin word *vacca* for cow), was subsequently adopted by Louis Pasteur for immunization against any disease. At the end of the nineteenth century, Shibasaburo Kitasato and Emil von Behring demonstrated the value of antitoxins in preventing disease by producing passive immunity to tetanus in animals that received graded injections of blood serum from another animal infected with the disease. This represented the first discovery of antibodies. Subsequently, Paul Ehrlich proposed his famous side-chain theory of immunity. He believed that toxic substances produced by bacteria bind to cells through side-chain molecular structures expressed on the cell surface, thereby causing disease, and that the body produces abundant side-chains (antibodies) in the blood to react with the specific bacterial toxins, thus preventing the toxins from reacting with the side-chains of cells. This theory exactly predicted the mechanism of antibody production from B cells in response to antigens. Around the same period, while studying starfish larvae, Elie Metchnikoff observed that certain white blood cells could engulf and destroy harmful bodies, such as bacteria. Metchnikoff designated these cells phagocytes and the process phagocytosis. This work shows the importance of innate immunity in the defence against microbial infection. Mechnikoff, together with Ehrlich, received the Nobel Prize for Medicine for the discovery of these two different aspects of immune responses, namely innate immunity and humoral (adaptive) immunity, respectively. However, humoral immunologists, including Behring, claimed that serum rather than cells destroyed the invading organisms and that innate immunity cannot explain the specificity of immunity. While humoral immunity research subsequently made remarkable progress, innate immunity was, until recently, regarded as an ancient and non-specific immunity that functions in the lower animal kingdom.

## 2. Innate immunity and adaptive immunity

Immune responses are largely categorized into innate immunity and adaptive immunity. Adaptive immunity is further divided into humoral immunity and cellular immunity. Humoral immunity is involved in the eradication of microbes present in the blood or fluid by generating antibodies, which are produced by B cells. On the other hand, cellular immunity is responsible for the eradication of cancer cells and microbes hidden inside cells, and is mediated by killer T cells. T and B cells express unique T-cell receptors (TCRs) and B-cell receptors (BCRs), respectively, and recognize a vast number of different antigens. TCRs and BCRs are generated by DNA recombination during the differentiation of T and B cells. Each TCR and BCR is composed of a variable region and a constant region. The variable region is encoded by different gene segments. Each member of the gene segment is randomly joined to the other members, resulting in the creation of a huge diversity of receptors. When a huge repertoire of TCRs and BCRs is generated in a ready-made manner, the repertoire includes receptors that react with components of the host. Lymphocytes harbouring self-reacting receptors are then excluded during differentiation. When a pathogen invades the body, T and B cells with the corresponding receptors are activated, and killer T cell development and antibody production are induced. At the same time, memory T and B cells are generated, allowing the host to respond more rapidly when the same pathogen invades the body again. Vaccination mimics an actual microbial infection in a highly attenuated condition, and its role is to generate memory cells against specific pathogens.

**Figure 1.**
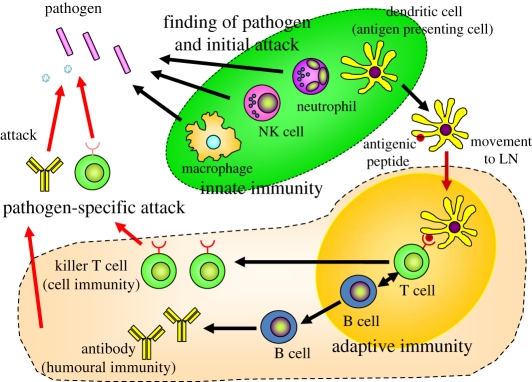
Interactions of innate and adaptive immunity. When pathogens invade the body, the innate immune system is activated first and participates in the initial attack against the pathogens. Among the cells involved in innate immunity, dendritic cells act as antigen-presenting cells and migrate from the infected tissue to the regional lymph nodes where they present the antigens to T cells. Subsequently, the adaptive immune system is activated, and antibody production and killer T cells are induced. The resulting antibodies and killer T cells specifically attack the pathogens.

On the other hand, innate immunity is mediated by leukocytes, macrophages and dendritic cells, which are collectively called phagocytes because they engulf and kill microbes. In the case of dendritic cells, they have an additional role of presenting antigenic peptides derived from microbes to T cells (figure 1). Until recently, far less attention had been paid to the study of innate immunity owing to its lack of specificity and diversity in microbe recognition. Innate immunity is found in all classes of plants and animals. For many years, innate immunity has been considered to be a remnant of the ancient host defence mechanism based on the phylogenetic development of the immune response. The cells of the innate immune system recognize, and respond to, pathogens in a non-specific way. However, unlike the adaptive immune system, they do not confer long-lasting immunity on the host. Although the innate immune system provides an immediate defence against infection, it has been considered to represent a temporary system until adaptive immune responses can be triggered. In higher organisms such as vertebrates, innate immunity is considered to have been replaced by adaptive immunity. However, recent studies have shown that the innate immune system possesses a greater degree of specificity than previously believed, and is highly developed in its ability to discriminate self from foreign pathogens. This discrimination relies to a great extent on a family of evolutionarily conserved receptors, designated Toll-like receptors (TLRs). Furthermore, there is accumulating evidence showing that activation of innate immunity is a prerequisite for the induction of acquired immunity. This paradigm shift is changing our thinking on the pathogenesis and treatment of infectious diseases, immune diseases, allergic diseases and cancers.

According to the original theory of the immune response, dendritic cells engulf invading pathogens, digest them into small peptides and express the peptide antigens on their cell surface. The dendritic cells then migrate from the infected tissue to the regional lymph nodes, where they present the antigens to naive T cells with the corresponding receptors. In this model, pathogen recognition takes place at the stage of T-cell activation in the lymph nodes.

On the other hand, in the new theory, in addition to the phagocytosis and digestion of pathogens, dendritic cells must be activated for T-cell activation. T cells are only activated when antigens are presented by activated dendritic cells. In normal tissues, cells die continuously, and the dead cells are removed by dendritic cells. Although the resulting self antigens are presented to T cells by the dendritic cells, the T cells do not become activated because the dendritic cells are not activated, thereby resulting in unresponsiveness or anergy. For efficient T-cell activation, dendritic cells must be activated, which is accompanied by the induction of costimulatory molecules and cytokine production. Such dendritic cell activation is mediated by TLRs.

Cancer immunotherapy has its roots in the work of Dr William B. Coley, an American surgeon who practised in New York. In the early 1880s, he noticed that cancers disappeared when patients experienced bacterial infections after surgery. Consequently, he started to treat patients by directly injecting live *Streptococcus pyogenes* bacteria, and later a mixture of dead *Streptococcus pyogenes* and dead *Serratia marcescens* bacteria, into inoperable tumours. Although his clinical trial achieved significant therapeutic effects, such treatments were not officially approved and became replaced by radiation therapy. Recent evidence has indicated that these substances induce anti-tumour immunity by activating TLRs on dendritic cells. Although dendritic cells engulf the dead tumour cells and present their antigens to T cells, anti-tumour immunity is not generated because the tumour cells are of self origin and cannot activate dendritic cells. However, the addition of microbial extracts containing TLR ligands enables tumour antigen presentation to T cells by activated dendritic cells, which results in the induction of effective anti-tumour immunity. A variety of bacterial extracts and synthetic compounds that activate TLRs are now undergoing clinical trials for a variety of cancers.

## 3. Toll-like receptors and their ligands

**Figure 2.**
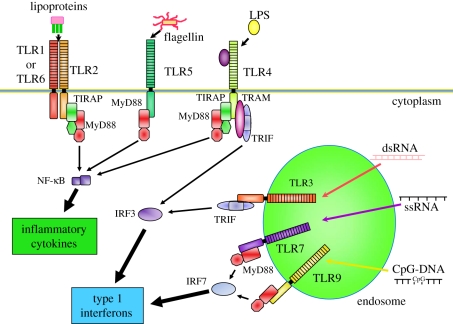
TLR ligands and signalling. TLR receptors recognize different microbial components: the heterodimer of TLR4 and MD-2 recognizes lipopolysaccharide (LPS); TLR2 recognizes triacyl and diacyl portions of lipoproteins together with TLR1 or TLR6, respectively; TLR5 recognizes flagellin, a major component of flagella; TLR3 recognizes double-stranded RNA; TLR7 recognizes single-stranded RNA and TLR9 recognizes bacterial and viral DNA, the so-called CpG DNA. The signalling pathways of TLRs are mediated by selective usage of adaptor molecules, MyD88, TRIF, TIRAP and TRAM. MyD88 is involved in all TLR signalling except for TLR3. TRIF is involved in TLR3 and TLR4 dependent activation of IRF3 via IKKi/TBK1, resulting in type 1 interferon production. TRAM is responsible for the TLR4-MyD88 dependent pathway involving recruitment of TRIF to the cytoplasmic portion of TLR4. TIRAP is involved in recruiting MyD88 to the cytoplasmic portions of TLR2 and TLR4. TLR7 and TLR9-dependent production of type 1 interferon requires direct interaction of MyD88 and IRF7, which occurs only in plasmacytoid dendritic cells. Nucleic acid-recognizing TLRs (TLR3, TLR7, TLR9) are present in the endosome.

TLRs are evolutionarily conserved between insects and vertebrates. Toll, as the founder member of the TLR family, was initially identified as an essential developmental protein for embryonic dorsoventral polarity in *Drosophila*, and was later shown to play a critical role in the anti-fungal response of flies [1]. To date, 12 members of the TLR family have been identified in mammals [2–4]. They are type 1 integral membrane glycoproteins, and structurally characterized by the presence of varying numbers of leucine-rich repeat (LRR) motifs in the extracellular portion as well as a cytoplasmic signalling domain homologous to that of the interleukin (IL)-1 receptor, designated the Toll/IL-1R (TIR) domain. The LRR domain is important for ligand binding. The LRR domains of the TLRs consist of 19–25 tandem copies of repeats that are 24–29 amino acids in length and contain xLxxLxLxx acid residues. Each unit consists of a β strand and an α-helix connected by loops. The LRR domains of the TLRs form a horseshoe structure and are directly involved in ligand recognition. TLRs can recognize a variety of components derived mainly from bacteria and viruses (figure 2). The TLR ligands can be categorized into lipid, protein and nucleic acid components. All TLR ligands are potent immune adjuvants that can trigger a vigorous immune response. Therefore, TLRs are also referred to as adjuvant receptors. The most potent and first identified ligand is lipopolysaccharide (LPS). LPS is found in the outer cell walls of Gram-negative bacteria and is recognized by TLR4 [5,6]. LPS is initially bound by a soluble factor, LPS-binding protein, in the serum and then transferred to target cells such as macrophages. Macrophages express a phosphatidylinositol-anchored cell surface molecule, cluster of differentiation 14 (CD14), which can capture and retain LPS, which then activates TLR4. A small secreted molecule, MD-2, is associated with TLR4 and critically involved in forming an LPS-recognizing complex [7].

Other lipid-containing components from the cell walls of a variety of micro-organisms are recognized by TLR2 and related TLRs, such as TLR1 and TLR6 [8–10]. Heterodimerization is critical for TLR2-mediated recognition. For example, TLR2 can recognize mycoplasma macrophage-activating lipopeptide-2 (MALP-2) when associated with TLR6. Meanwhile, a TLR2/TLR1 heterodimer is involved in recognizing bacterial lipopeptide (BLP). MALP-2 and BLP carry a diacylated and triacylated cysteine residue at their N-terminus, respectively, and this subtle difference is discriminated by the TLR2-containing heterodimers. These receptors recognizing cell wall components are expressed not only in immune cells but also in non-immune cells, including fibroblasts, endothelial cells, adipocytes, epithelial cells and glial cells.

In addition to cell wall components, several other components including bacterial DNA and flagellin have immunostimulatory activity. TLR5 is involved in recognizing flagellin, a component of the bacterial flagella responsible for motility in a liquid [11]. Flagellin can elicit mucosal immune responses by acting on epithelial cells or macrophages. Although flagellin is a protein, its amino acid structure is highly conserved, suggesting that it acts as a target for innate immune recognition. TLR5 expression is observed around the body parts that are prone to invasion by flagellated bacteria, such as the digestive tract, urinary tract and respiratory tract. In the case of the intestinal epithelia, TLR5 expression is confined to the basolateral face and not detected on the apical face. Therefore, TLR5 only becomes activated when flagellated bacteria invade the body, such that chemokines are produced in the epithelium and leukocytes are accumulated, thereby causing inflammatory responses. TLR5 is also expressed in dendritic cells residing in the mucosa [12].

Nucleic acids are also recognized by TLRs. Bacterial DNA has long been known to act as a strong immune adjuvant. This adjuvant activity is dependent on unmethylated CpG motifs. CpG motifs are more abundant in bacterial DNA than in mammalian DNA. Furthermore, bacterial CpG is unmethylated whereas mammalian CpG DNA is methylated. Therefore, unmethylated CpG DNA can be regarded as non-self and is recognized by TLR9 [13]. Viral DNA is also rich in CpG motifs and DNA virus infection triggers TLR9 signalling.

Several small synthetic molecules, including imidazoquinoline derivatives and several anti-cancer drugs, are well known for their antiviral activity. This activity is dependent on TLR7 [14]. Drugs like imidazoquinoline have nucleic acid-like structures, suggesting the possibility that TLR7 is involved in the recognition of viral RNA. Indeed, single-stranded RNA was found to be a ligand for TLR7 and also for its close relative, TLR8 [15,16]. These interactions are critical for sensing RNA virus infection. RNA virus infection also induces the production of double-stranded RNA (dsRNA) in infected cells, and these dsRNAs can act as immune adjuvants after recognition by TLR3 [17].

Importantly, TLR2 and TLR4 are mainly expressed on the plasma membrane, while the nucleic acid-recognizing TLRs are expressed in the endosome. The latter is understandable because nucleic acids are embedded inside the pathogens. In the endosome, the nucleic acids are released from the virus or virus-infected cells and encounter their respective TLRs. Consistent with this, nucleic acid-recognizing TLRs are expressed mainly in phagocytes, such as macrophages and dendritic cells.

## 4. Myd88-dependent and -independent pathways in toll-like receptor signalling

TLRs recognize different microbial components. Similarly, the signalling pathways of individual TLRs differ from one another. Initially, the signalling pathways of the TLRs were thought to be identical, and to be entirely dependent on an adaptor molecule named MyD88. However, we noted the presence of an MyD88-independent pathway because we found that MyD88-deficient cells could still activate nuclear factor kappa-light-chain-enhancer of activated B cells (NF-κB) in response to LPS [18]. Once the ligands of the individual TLRs had been identified, stimulation of cells with the different TLR ligands was found to induce distinct patterns of gene expression, indicating differences in the signalling pathways among the TLRs. Subsequent studies showed that the differences in the signalling pathways among TLRs arise through selective usage of adaptor molecules [19,20] (figure 2). MyD88 is essential for all TLR signalling pathways except the TLR3 signalling pathway. MyD88 activates NF-κB via the IL-1 receptor-associated kinases and TRAF6, resulting in the production of inflammatory cytokines. Therefore, the MyD88-dependent pathway is responsible for inflammatory reactions. In addition to the MyD88-dependent pathway, there is an MyD88-independent pathway for TLR3 and TLR4 signalling. The MyD88-independent pathway is regulated by another adaptor molecule named TRIF [21]. TRIF activates the transcription factor interferon regulatory factor 3 (IRF3), which becomes phosphorylated and translocates from the cytoplasm to the nucleus, resulting in the production of type 1 interferons (IFNs). Therefore, the TRIF-dependent pathway is involved in antiviral responses. TANK-binding kinase 1 (TBK1) and IkB kinase, inducible (IKKi) were shown to act as IRF3 kinases. TLR7 and TLR9 recognize viral components, and induce the production of type 1 IFNs. However, the molecular mechanism is quite different from that of TLR3- and TLR4-dependent type 1 IFN production. MyD88 is essential for type 1 IFN production through TLR7 and TLR9. In various cell types, MyD88-dependent signalling is associated with inflammatory responses via the activation of NF-κB and mitogen activated (MAP) kinases. In plasmacytoid dendritic cells (pDCs) only, TLR7 or TLR9 activation induces the production of type 1 IFNs via the direct association of MyD88 with IRF7, independently of TBK1 and IKKi, in contrast to the case for TRIF-dependent type 1 IFN production [22].

## 5. Cytoplasmic helicases for sensing of viral infection

**Figure 3.**
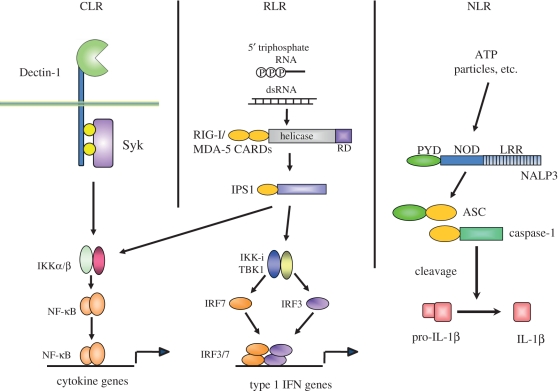
C-type lectins, RIG-like receptors (RLRs) and NOD-like receptors. C-type lectins (CLRs) recognize carbohydrates on micro-organisms via the carbohydrate-binding domain. Dectin-1, Dectin-2, Mincle and CLEC9A are well studied. For example, Dectin-1 activates the Syk tyrosine kinase via the immunoreceptor tyrosine-based activation motif (ITAM) domain, resulting in production of inflammatory cytokines. RLRs comprise RIG-I and MDA5. RLRs are composed of two N-terminal caspase-recruitment domains (CARDs), a central DEAD box helicase/ATPase domain, and a C-terminal regulatory domain. They are localized in the cytoplasm and recognize the genomic RNA of dsRNA viruses, and dsRNA generated as the replication intermediate of ssRNA viruses. RLRs interact with IPS1 via their CARD domains, resulting in type 1 interferon production through IKKi/TBK1. NLRs are composed of a central nucleotide-binding domain (NOD) and C-terminal leucine-rich repeats (LRRs). The N-terminal portions harbour protein-binding motifs. NODs activate caspase-1, resulting in processing of pro-IL-1β to mature IL-1β.

Although the involvement of TLRs in virus-induced cytokine and IFN production is well established, fibroblasts lacking both MyD88 and TRIF are still capable of expressing IFN-inducible genes in response to RNA virus infection, indicating the existence of TLR-independent virus detectors [23,24] (figure 3).

An RNA helicase, retinoic acid-inducible gene I (RIG-I), was identified as a cytosolic sensor for viral invasion, and induces type 1 IFNs in a TLR-independent manner [25,26]. Melanoma differentiation-associated gene 5 (MDA5) is homologous to RIG-I. Both helicases possess two N-terminal caspase-recruitment domains (CARDs) followed by an RNA helicase domain. The CARDs are responsible for signal transduction, which leads to the activation of NF-κB and IRF3/7 via their adaptor molecule, IFN-β promoter stimulator 1 (IPS-1; also called Cardif, MAVS or VISA), which is located on the outer membrane of mitochondria [27,28]. TBK1 and IKKi are also responsible for the activation of RIG-I- or MDA5-dependent IFN production, indicating that the signalling pathways triggered by TLR stimulation and RIG-I converge at the level of TBK1/IKKi. The functional and differential roles of RIG-I and MDA5 were revealed by gene targeting. RIG-I detects a variety of RNA viruses by recognizing 5′-triphosphate short dsRNAs or long dsRNAs (several hundred to several thousand base pairs). On the other hand, MDA5 detects picornavirus family members by recognizing long dsRNAs of more than 2 kbp [29,30]. RIG-I and MDA5 are ubiquitously expressed in all cells. DNA viruses and dsDNAs are also recognized in the cytoplasm. Although several candidates for dsDNA sensors have been reported, essential roles of these receptors have not been confirmed by gene targeting and the initial dsDNA recognition mechanism remains unclear. Recently, stimulator of interferon genes (STING) was shown to be a mediator of dsDNA-induced type 1 IFN production [31]. STING is a multi-spanning membrane protein that associates with TBK1. STING-deficient cells are hyporesponsive to dsDNA.

## 6. Nucleotide-binding domain-like receptors and C-type lectin receptors

Bacteria are also detected in the cytoplasm by the members of the nucleotide-binding domain (NOD)-like receptor (NLR) family (figure 3). NLRs consist of a C-terminal LLR domain, a central NOD and an N-terminal effector domain that initiates signalling. The minimal components of peptidoglycan are recognized by NOD1 and NOD2, leading to NF-κB activation and inflammatory response induction [32]. In addition to NOD1 and NOD2, several NLRs are present in the cytoplasm. These NLRs are involved in inflammasome formation and the production of mature IL-1 and IL-18 [33]. The activities of IL-1β and IL-18 are regulated at both the transcriptional and post-translational levels. Transcriptional activation of these genes leads to the production of pro-IL-1β and pro-IL-18. These proteins are present in the cytoplasm in inactive forms. Caspase-1 activation is required for the generation of mature IL-1β and IL-18, both of which are secreted from the cytoplasm to the outside of the cell. Inflammasome formation is essential for caspase-1 activation. At present, four types of inflammasome have been identified, namely nucleotide-binding domain and leucine-rich repeat containing family, pyrin domain containing 1 (NLRP1), nucleotide-binding domain and leucine-rich repeat containing family, CARD domain containing 4 (NLRC4), NLRP3 and absent in melanoma 2 (AIM2) inflammasomes, although AIM2 is not a member of the NOD family. Flagellin (TLR5 ligand) and imidazoquinoline/RNA (TLR7 ligands) activate NLRC4 and NLRP3 inflammasomes, respectively. In addition, several crystallized molecules, such as monosodium urate, asbestos and cholesterol crystals, are recognized in the cytoplasm via the formation of NLRP3 inflammasomes. The flagellin monomer is shaped like the capital Greek gamma (*Γ*) and is formed by domains D0 through D3. Interestingly, different parts of flagellin are recognized by TLR5 and NLRC4 inflammasomes, since the D1 domain is recognized by TLR5 and the D0 domain is responsible for NLRC inflammasome activation.

β-Glucan is the most abundant cell wall component of fungi and yeasts. It is also present in bacteria and plants, but is not found in animals. β-Glucan is recognized by a C-type lectin receptor (CLR), Dectin-1. Dectin-1 consists of an extracellular carbohydrate-recognizing C-type lectin domain (CTLD) and a cytoplasmic tyrosine-containing domain that is similar to the immunoreceptor tyrosine-based activation motif (ITAM; figure 3). Engagement of Dectin-1 by β-glucan triggers the recruitment of spleen tyrosine kinase (Syk), which activates NF-κB through CARD9, Bcl10 and MALT1 as well as caspase-1 [34]. Dectin-1 recognizes several fungal species, including a number of human pathogens such as *Candida*, *Aspergillus*, *Pneumocystis* and *Coccidioides*, and plays an important role in the host defence against these pathogens. Another CLR, Dectin-2, is predominantly expressed on tissue macrophages, dendritic cells and inflammatory monocytes, and possesses a classical sugar-binding CTLD that recognizes high-mannose structures in a Ca^2+^-dependent manner. Using this domain, Dectin-2 recognizes a variety of pathogens including capsule-deficient *Cryptococcus neoformans*, *Candida albicans*, *Saccharomyces cerevisiae*, *Mycobacterium tuberculosis*, *Microsporum audounii*, *Trichophyton rubrum*, *Paracoccidioides brasiliensis* and *Histoplasma capsulatum*. Although it has a short cytoplasmic tail that lacks traditional signalling motifs, Dectin-2 associates with the ITAM-containing FcR*γ* adaptor and can trigger intracellular signalling through the Syk/CARD9 pathway to induce a variety of cellular responses. Macrophage-inducible C-type lectin (Mincle) is primarily expressed by activated macrophages. Similar to Dectin-2, Mincle possesses a single extracellular CTLD and a short cytoplasmic tail, and associates with the adaptor FcR*γ* to trigger intracellular signalling through the Syk/CARD9 pathway. Mincle recognizes a variety of endogenous and exogenous ligands, such as necrotic cells (small nuclear ribonucleoprotein SAP130), mycobacteria (trehalose dimycolate or mycobacterial cord factor) and certain fungi (α-mannan), including *Candida*, *Saccharomyces* and *Malassezia*.

## 7. Influenza virus and immunity

Recent advances in our understanding of innate immunity have revealed that activation of the innate immune system is essential for subsequent adaptive immune responses including specific antibody production and cytotoxic T-lymphocyte activation, which play key roles in the protection against virus infection.

**Figure 4.**
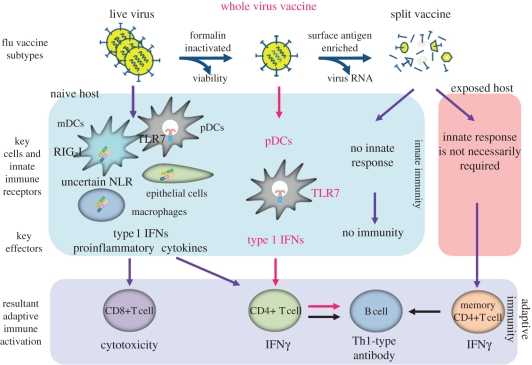
Induction of adaptive immunity by influenza viral infection and vaccination. In the case of live influenza viruses, the viruses infect a variety of cells except for plasmacytoid dendritic cells (pDC) and produce RNAs, which are recognized by RIG-I, resulting in type 1 interferon production. pDCs are resistant to infection and phagocytose the viral particle, and RNA is liberated in the phagolysome, which result in activation of TLR7/MyD88-dependent pathway and type 1 interferon production. In the case of inactivated whole virus vaccine, this cannot infect but pDCs engulf the inactivated virus, and subsequent activation of TLR7/MyD88-dependent pathway leads to production of type 1 interferon. This pathway is also essential to induction of adaptive immunity against influenza virus. Split vaccine lacking RNA is not effective in naive persons but can play some role in protection by activating memory B cells in people who have already experienced influenza viral infection.

In the case of influenza virus infection or vaccination with an attenuated live virus, the RIG-I/IPS-1 and TLR7/MyD88 pathways are triggered in tissue-specific manners. Specifically, the RIG-I/IPS-1 pathway is activated in many cell types, but not in pDCs, while the TLR7/MyD88 pathway induces type 1 IFN production only in pDCs. In live virus infection, many cells are infected, viral replication takes place, and RNA is produced in the cytoplasm and recognized by RIG-I. Although pDCs are resistant to infection, the viral particles are incorporated into the endosome/lysozome, where they are disrupted, leading to liberation of the RNA and its recognition by TLR7. On the other hand, the inactivated influenza virus used as a vaccine cannot infect cells. Nevertheless, pDCs phagocytose the viral particles and type 1 IFNs are produced in a TLR7/MyD88-dependent manner [35] (figure 4). There is a tendency to use purified components from influenza virus particles (subunit vaccines) as vaccines to avoid adverse effects. A subunit vaccine, Split HA, is now used for seasonal flu in Japan. However, this product lacks influenza virus-derived RNA and does not induce innate immune responses, and therefore, could not protect naive mice that had never experienced influenza virus infection [36]. The Split HA vaccine may be useful for adult humans who have experienced repeated flu infections and have memory T and B cells. When the Split HA vaccine was injected together with CpG DNA, this formulation induced both innate and adaptive immunity against influenza virus in a TLR9-dependent manner. These findings reinforce the notion that efficient induction of adaptive immunity requires immunostimulatory TLR ligands as adjuvants in addition to antigens (figure 4).

## 8. Conclusions

Innate immunity is essential for both the development and modulation of adaptive immunity. Pathogens are recognized by four types of innate immune-associated receptors, namely TLRs, NLRs, RIG-like receptors and CLRs. The initial recognition of pathogens by these receptors induces inflammatory reactions at the infected site, and also triggers adaptive immunity against the pathogens. We need to clarify more precisely the immune response pathway, starting from the activation of innate pathogen receptors, that finally leads to the development of protective immunity against individual pathogens. Such findings will enable us to develop rational vaccines.
